# Books

**Published:** 1883-12

**Authors:** 


### Books.

---

INSANITY CONSIDERED IN ITS MEDICO-LEGAL RELATIONS, by T. R. Buckham, A.M., M.D.; J B Lippincott & Co., per S. P. Richards & Son, Atlanta, Ga, 1883 was merely mentioned in our last number.

This able and well-timed treatise deserves a longer and more careful notice than can be given it here. The chief object in view of the author is to show the fearful uncertainty of verdicts in trials involving insanity.

Properly comprehending the impossibility of recognizing insanity when present, in any case, or of treating it upon any rational grounds, when discovered, without a rational theory as to what is insanity, the author devotes a large part of his book to the discussion of the four prominent theories, or hypotheses, as the case may be, which are now dividing the world on this great subject—that is, rather, what mind is, and how is it affected, viz: the physical media theory, the metaphysical or psychical theory, the somatic theory, and the intermediate hypothesis of Wharton and Stille.

He overthrows the latter hypothesis (which is but a hypothesis), shows the absurdity and impossibility of the metaphysical and somatic theories, and establishes the physical media theory by a course of reasoning apparently impregnable. It is only necessary to state the old somatic proposition, so often resurrected and rehabilitated to suit the skepticism of the age, to refute it to a sound understanding: Man is an automaton; has no free will, only the inevitable inclinations given him by heredity. Its ablest advocate, Dr. Mandsley, in his "Body and Mind," thus cogitates: "At the end of all the most subtile and elaborate disquisitions concerning moral freedom and responsibility, the stern fact remains that the inheritance of a man's descent weighs on him through life as a good or bad fate. How can he escape from his ancestors? Stored up mysteriously in the nature which they transmit to him, he inherits not only the organized results of the acquisitions and evolution of generations of men, but he inherits also certain individual peculiarities or proclivities which *determine irresistibly the general aim of his career.* While he fancies that he is steering himself and determining his course at will, *his character is his destiny.* The laws of hereditary transmission are charged with the destinies of mankind—of the race and of the individual."

As our author says, this is outspoken, flat-footed human irresponsibility doctrine. The establishment of this doctrine would obviously settle forever all possibility of a vexed question relating under any circumstances to human guilt. Man is not a free-agent. The only sensible inquiry would be, is he so far beside himself as to be dangerous if suffered to go at large?

On the other hand, it is only necessary to give expression to the physical media theory in order to show its claims upon the world's belief. "The physical media theory," like the "metaphysical theory," regards the mind as a distinct incorporeal entity, not dependent upon the body for its existence; but, unlike the "metaphysical theory," it recognizes the most intimate relations between mind and body, and holds that in this life mind is *wholly dependent* for the manifestations of its operations on certain organs of the body which we designate "physical media."

His theory of mind being true, the definition given by the author of insanity is the best that has yet appeared: "Insanity is a diseased or disordered condition, or malformation of the physical organs through which the mind receives impressions or manifests its operations, by which the will and judgment are impaired and the conduct considered irrational." And, therefore, "insanity being the result of physical disease, *it is a matter of fact* to be determined by medical experts, *not a matter of law* to be decided *by legal tests and maxims.*"

After giving a fearful array of various and contradictory opinions of the judges, jurists and legal authors of England and United States, in relation to the legal tests or criteria of insanity, the author asks: "With such variable criteria in insanity trials, is it surprising that there should be uncertainty of verdict." Quite one half of the book is devoted to the discussion of "experts in insanity" in which ample and pertinent illustration is drawn from the famous trial of Guiteau.

We can not close this brief review of this valuable contribution to medico-legal literature, without a suitable reference to the author's sound opinions in reference to the moral and legal responsibilities of the drunkard. It has got to be quite the fashion for certain, flippant, shallow writers to babble about the hereditary, irresponsible character of the inebriate. The author boldly and justly assumes the contrary. "As a general rule," he says, "even *voluntarily* induced *mania a potu* should no more relieve from responsibility than voluntarily induced drunkenness."

This work of Dr. Buckham is able, sound and most timely; judges, jurists, physicians and legislators will consult not only their own interest but that of their country, by a careful study of its teachings. The superior paper, and beautiful typography of the book will attract the attention of even the casual reader.

---

THE ROLLER BANDAGE, by W. B. Hopkins, M. D., Philadelphia; J. B. Lippincott & Co., 1883, through S. P. Richards & Son, Atlanta, Ga.

This is a primer to show, as it were at a glance the method of applying the roller bandage under all circumstances and in every possibly locality. This is accomplished by a series of illustrations photographed on the living subject, in all its known points of difficulty and complexity. The student will need no better teacher than the lucid directions of this little book. We heartily commend it.

---

CHEMISTRY: ORGANIC AND INORGANIC, WITH EXPERIMENTS, by Charles London Bloxam, Prof. of Chemistry in King's College, London, &c. From the Fifth Revised English Edition, with 292 Illustrations. Philadelphia, Henry C. Lea's Son & Co., 1883.

This great treatise on chemistry by the distinguished author has been a standard work both in England and America. The fact that so expensive a scientific book should have reached its fifth edition at home, with a republication in America, is sufficient guaranty of its value. As a text-book for reference, alike to professors, practical chemists and students, we know no more reliable and copious work than this.

It presents several admirable features which may be mentioned in detail. First, avoiding, as far as possible, technicalities, the author gradually introduces the student to the more complex principles and methods of the science. He is not perplexed and disgusted at the threshold of his course by an incomprehensible terminology, and tedious array of scientific abstractions. Second, the educational experiments of the author are just such, as a long experience in the lecture room has taught him to be most useful and available to the student. The experiments are simple and inexpensive, and the apparatus and manipulations so represented by wood engravings, in each case as to fix them at once, in the understanding and memory of the pupil.

Third, it is not the least valuable feature in the book, that English weights and measures, and Fahrenheit's thermometer are, alone, employed. Not that there are not many admirable advantages in the centigrade thermometer and the Metric system, but like the people of Great Britain, we are yet an English speaking people, and the day far away in the indefinite uncertain future, when Celsius' thermometer and the French system of weights shall come into general use. It is not yet universal even in France. The work, contains, also, a special department, devoted to Metallurgy and some other branches of applied chemistry, very useful and valuable to such as are being educated for employment in manufactories.

There are other improvements and additions to this revised edition of the author's great work, but we have not the space to notice them. We predict a large run for this admirable treatise on chemistry in the United States.

---

HANDBOOK OF ELECTRO-THERAPEUTICS. By Dr. Wilhelm Erb, Prof. in the University of Leipzig. Translated by L. Putzel, M. D., Neurologist to Randall's Island Hospital, etc. With 39 Wood Cuts. New York. Wm. Wood & Co., 1883.

This is the June number of Wood's Library of Standard Medical Authors. Part I, Lecture I, discusses the following subjects under the general head of Physical Introduction—various kinds of currents—contact electricity—the galvanic current—galvanic currents—induction electricity—the Faradic current—the ordinary apparatus and auxiliary apparatus—induction apparatus and galvanic batteries.

Lecture II—accessory apparatus :—selector or element numerator—polarity changes—galvanometer—measurement of the absolute strength of the current—rheostat—conducting wires—electrodes of their various forms—electrical table—physical and physiological recognition of the poles, etc , etc.

The larger part of the work is, of course, taken up with the great subjects of electro-diagnosis and electro-therapeutics. And we risk nothing in the assertion, that no book has yet been published in the United States so replete in practical and scientific information on electro-therapeutics—its principles, apparatus and applications, as this great standard work of Professor Erb.

Index to the Transactions of the American Medical Association. Vols. I–XXXIII. Prepared by Wm. B. Atkinson, M.D., Permanent Secretary.

---

A Treatise on Syphilis in New Born Children and Infants at the Breast, by P. Diday, Ex-Surgeon to the Hospital De L'antiquaille Lyons, translated by G. Whitley, M. D., with Notes and an Appendix by F. R. Sturgis, M. D., Prof. of Venereal and Skin Diseases in the New York Post Graduate Medical College &c., N. Y., William Wood & Co., 1883.

From the days of record, French teachers and writers have stood foremost in the intricate, well-worn field of the venereal. This fresh contribution by M. Diday, adds another score to their fame.

We have examined no work, recently, in so small a compass—so unpretentious—that contains so much valuable, practical and scientific information on the subject it undertakes to teach, as this admirable manual, the October number of Wood's Library of Standard Medical Authors.

The translation by Dr. Whitley is sufficiently lucid and idiomatic; the reader, unless reminded by the title page, would scarcely recognize the Gallic atmosphere, in which the book first saw the light. Dr. Sturgis' annotations give an additional value to the work enriching and rounding it up with contributions and suggestions from American experience of high and most trustworthy authority.

It will be sufficient in order to apprise medical men and students of the great practical value of this work, to simply give a few topics from its chapter of contents: Part I, is devoted to Etiology—historical notice—Congenital Syphilis—influence of the father—influence of the mother—influence of the mother infected before the moment of conception—influence of the mother infected after conception—combined influence of both parents.

Acquired Syphilis—infection during labor—infection by lactation by a morbid lesion in the nurse by the milk, etc.

Part II, contains detailed and diagnostic indications of each lesion known to the disease.

Part III, Etiological and Semeilogical Prognosis.

Part IV, Medico-Legal Bearings. The contents of this chapter is of great practical value both to physicians and lawyers.

Part V. The treatment.—This alone is worth the price of the book.

---

CLINICAL CHEMISTRY: AN ACCOUNT OF THE ANALYSIS OF BLOOD, URINE, MORBID PRODUCTS, &C., WITH AN EXPLANATION OF SOME OF THE CHEMICAL CHANGES THAT OCCUR IN THE BODY IN DISEASE, by Charles H. Ralfe, A. M, M D., Fellow of the Royal College of Physicians, London, etc. Illustrated with 16 engravings. Henry C Lea's Son & Co., Philadelphia, Pa.

This is a small book, but appears to contain a great deal in a small space. As its title implies it has been written for a practical purpose, viz: to furnish student and practitioners with a concise description of the best methods of examining chemically abnormal blood, urine, morbid products, etc., at the bedside or in the hospital laboratory. It was purposely made brief and simple. We heartily commend it to students and practitioners.

MANUAL OF GENERAL MEDICAL TECHNOLOGY, INCLUDING PRESCRIPTION WRITING. By Edward Curtis, A. M., M. D., Prof. of Materia Medica and Therapeutics, College of Physicians and Surgeons, etc., New York City. Wm Wood & Co, 1883.

This is one of Wood's pocket manuals, an excellent little companion that is very much needed by the profession, both students and old practitioners, if there is anything suggestive in the files of written prescriptions found in our drug stores.

It would be well for the people, and, perhaps, for the profession, to publish one of these files each year from every leading drug store with the names in full of the writers. The children would have puzzles to work at, and to send to literary and religious journals, to their hearts' content.

---

EPITOME OF SKIN DISEASES, WITH FORMULÆ FOR STUDENTS AND PRACTITIONERS. By the late Tilbury Fox, M.D., M.R.C.P. Third American Edition, Revised and with Additions. By T. Colcott Fox, B.A., (canab), M.B., (London). Philadelphia: Henry C. Lea's Son & Co. 1883

The favorable reception accorded to this book on both sides of the Atlantic would seem to show that it has realized the object for which it was prepared.

---

MEDICAL ELECTRICITY: A PRACTICAL TREATISE ON THE APPLIANCES OF ELECTRICITY TO MEDICINE AND SURGERY. By Roberts Bartholow, A.M., M.D., LL.D, Professor of Materia Medica and General Therapeutics in Jefferson Medical College, of Philadelphia, etc., etc., with ninety-six illustrations. Philadelphia: Henry C. Lea's Son & Co.

We lack space to give this work such notice as it merits. The author's name alone will go far in recommending it to the profession and to all who may be interested in this new but rapidly enlarging subject of therapeutical research and application.

---

A PRACTICAL TREATISE ON MATERIA MEDICA AND THERAPEUTICS. By Roberts Bartholow, M. A, M. D., L. L D. Prof Materia Medica and Gen. Therapeutics in Jefferson Med. Col, Philadelphia. Fifth Edition Revised and Enlarged. New York: D. Appleton & Co., Bond Street, 1884 Per S P. Richards & Son, Atlanta, Ga.

This great work of Dr. Bartholow deserves a longer and more careful notice than we now have space to devote to it. He will review it in our next number.

A Treatise on Bright's Disease of the Kidneys: Its Pathology, Diagnosis and Treatment, with Chapters on the Anatomy of the Kidney, Albuminuria, and the Urinary Secretion. By Henry B. Millard, M.D., A.M., with numerous original illustrations. New York: Wm. Wood & Co. 1884.

This admirable work is the outgrowth of nearly twenty-six years of hospital experience, of an extensive private practice, and of several years' study in the laboratory of pathological and healthy kidneys of men and animals. The illustrations are unusually clear and instructive, whilst in the binding, material and typography of the book we are reminded of the excellence of old English works. We heartily commend this treatise to the profession on one of the most obscure, perplexing and intractible diseases to which humanity is heir.

